# *Citrus aurantium* L. and *Citrus latifolia* extracts as alternative control agents for *Aedes aegypti* (Diptera: Culicidae)

**DOI:** 10.1186/s40659-025-00600-x

**Published:** 2025-07-02

**Authors:** Andrea Martínez Gordon, Alejandro Figueredo López, Ingrid Dayana Jiménez, Laura Barrera Martínez, Oscar H. Pardo Cuervo, Nidya Alexandra Segura Guerrero

**Affiliations:** 1 Grupo de Investigación en Ciencias Biomédicas UPTC/GICBUPTC, Universidad Pedagógica y Tecnológica de Colombia - UPTC, Tunja, Boyacá Colombia; 2 Grupo de Investigación Catálisis, Universidad Pedagógica y Tecnológica de Colombia - UPTC, Tunja, Boyacá Colombia

**Keywords:** *Aedes aegypti*, Chloroform extracts, *Citrus aurantium*, *Citrus latifolia*, Chemical composition, Mosquito control

## Abstract

**Background:**

*Aedes aegypti* is a vector of arboviral diseases. Their control has traditionally relied on the use of chemical insecticides; however, this strategy has failed. As an alternative, the use of natural products with a high content of biologically active compounds has been evaluated for the control of this mosquito. It is well known that citrus fruits contain compounds with insecticidal activity. For this reason, and considering the high production of *Citrus aurantium* L. and *Citrus latifolia* Tanaka ex Q. Jiménez in Colombia, the aim of this research was to establish the susceptibility of *Ae. aegypti* to extracts from the exocarp of these two citrus species as an environmentally sustainable approach to vector control.

**Methods:**

Following WHO methodology, the adulticidal activity of exocarp extracts of *Citrus aurantium* L. and *Citrus latifolia* Tanaka ex Q. Jiménez strain Moniquirá, obtained with ethanol, *n*-hexane, or chloroform by reflux for 4–6 h, was evaluated. The chemical composition of the extracts was established using GC-MS.

**Results:**

100% mortality was achieved with the ethanolic extract of *C. aurantium* and the chloroform extract of *C. latifolia* obtained during 6 h of extraction, with concentrations of 90 and 120 mg/mL at 12 and 6 h p.e. respectively. Highly significant differences (*p* < 0.001) were determined in concentration, type of solvent, and extraction time regarding *Ae. aegypti* mortality for the two species. For the 6 h ethanol extract of *C. aurantium*, the LC_50_ was 32.2 mg/mL after 12 h p.e, while for the 6 h chloroform extract of *C. latifolia*, the LC_50_ was 9 mg/mL after 6 h p.e. The composition of the chloroform extracts is similar, but the concentration of most compounds increased in the 6 h extract. Sabinene, β-Pinene, R-limonene and γ-terpinene were the major components.

**Conclusions:**

The chemical composition of the extracts showed variable concentrations depending on the extraction time. Indeed, the chloroform extracts of *C. latifolia* exocarps obtained by reflux at 6 h showed the better potential as control agents for *Aedes aegypti.* These results form the basis for the future development of a natural product that can be used by residents of endemic areas to *Ae. aegypti.*

## Background

The mosquito *Aedes aegypti* has a widespread distribution in tropical and subtropical regions worldwide [1, 2]. *Ae. aegypti* is one of the most important vectors in public health primarily responsible for transmitting Flaviviruses, including dengue virus (DENV), Zika virus (ZIKV), and yellow fever virus (YFV), and Alphaviruses, such as Chikungunya virus (CHIKV). These arboviruses cause diseases in humans, representing a significant global threat.

Globally, dengue virus has a significant impact, with an estimated 390 million infections occurring annually, including 96 million symptomatic cases [3]. In Colombia, 131,784 cases of dengue were reported between January to December 2023, showing an increase of 63,658 cases compared to the same period in 2022, and triggering ongoing epidemiological alerts in the country. By epidemiological week 29 of 2024, 23,920 cases had been recorded, a threefold increase from the same period the previous year [4–6]. These statistics highlight the significant challenge faced by public health systems both globally and nationally, underscoring the importance of new effective strategies for dengue prevention and control. This issue represents a significant burden for the medical care of affected populations and also contributes to social problems, such as increased poverty, loss of economic activities, and school absenteeism [7]. Additionally, traditional control measures can lead to the development of genetic resistance in mosquito vector populations [8–10].

To date, there are no effective antiviral treatments against the aforementioned arboviruses. However, a vaccine against DENV, Dengvaxia^®^ (CYD-TDV), has been approved. Despite this, it was found that seronegative individuals immunized with this vaccine faced an increased risk of developing severe dengue cases [11]. On the other hand, the attenuated vaccine TAK-003 has shown promising results [12]. Consequently, health entities mainly focus on vector control through the continuous use of insecticides, which, although they can control some vector populations, cause negative environmental impacts by contributing to increased air pollution, water sources contamination, and soil pollution. They also affect a wide diversity of non-target species, including humans [13, 14]. Other strategies to control the spread of *Ae. aegypti* include environmental management practices involving the use of natural enemies such as parasites [15, 16] and predators [17–20], genetic interventions to reduce both their reproduction and viral replication [21–24].

Natural extracts represent a safer and environmentally friendly alternative for vector control compared to synthetic insecticides, as these extracts are biodegradable and have minimal negative effects on human health [25, 26]. In particular, citrus extracts have demonstrated insecticidal properties against both adult and larval *Ae. aegypti*, thanks to the presence of chemical compounds such as limonoids, flavonoids, and citronellal, which are considered responsible for their natural repellent and insecticidal activity against the vector [27–30].

Concurrently, Colombia recorded a total citrus production of 1.45 million tons, of which 47% were oranges, 27% mandarins, and 26% lemons [31]. The Boyacá region in Colombia stands out as one of the main producers of these fruits due to its favorable environmental conditions [32]. This abundance creates an opportunity to utilize citrus waste as raw material in the development of bioproducts aimed at controlling *Ae. aegypti* mosquitoes.

In this context, although some studies have explored the efficacy of plant extracts in reducing *Ae. aegypti* mosquito populations [33, 34], to our knowledge, the efficiency of citrus extracts as adulticides has not been evaluated. Therefore, the aim of this research was to establish the susceptibility of *Ae. aegypti* to extracts from the exocarp of Bitter Orange (*Citrus aurantium* L.) and Tahitian Lemon (*Citrus latifolia* Tanaka ex Q. Jiménez), as an environmentally sustainable alternative for vector control.

## Materials and methods

### Collection of plant materials

Taxonomic verification of the plant species was performed at the Universidad Pedagógica y Tecnológica de Colombia Herbarium in Tunja, Colombia. Ripe fruits of *Citrus aurantium* L. and *Citrus latifolia* Tanaka ex Q. Jiménez were acquired in the municipality of Moniquirá, Colombia (5°52′28″N, 73°34′18″W). The selection of fruits was carried out following the methodology proposed by Gupta [35] which involves careful observation of color, prioritizing fruits with specific hues indicating their ripeness. Additionally, firmness to the touch was evaluated, opting for fruits with a firm texture but not excessively rigid.

### Extraction process

To determine the effect of extraction time on the concentration of biologically active products, and based on previous studies [36] extraction times of 4 and 6 h were selected. Similarly, to evaluate the effect of solvent polarity on affinity to extract biologically active products, ethanol, chloroform, and hexane were chosen, as they exhibit high, medium, and low polarity, respectively. Briefly, fragments of exocarp measuring 1 cm² were dried at 60 °C for 24 h and then ground until uniform particle size was achieved. Subsequently, 20 g of exocarp were mixed with 100 mL of solvent (96% ethanol, n-hexane, or chloroform) and heated to reflux temperature for 4–6 h. After reaching room temperature, the mixture was filtered. Finally, the solvent was removed by reduced pressure distillation using a VIRESA^®^ rotary evaporator. The resulting extract was coded (Table 1), stored at 4 °C, and protected from light until use.

**Table 1:** Codes of evaluated extracts

Citric	Extraction time (h)	Solvent		
		Chloroform	Ethanol	*n*-Hexane
*C. aurantium*	4	AuCl4	AuE4	AuH4
	6	AuCl6	AuE6	AuH6
*C. latifolia*	4	LaCl4	LaE4	LaH4
	6	LaCl6	LaE6	LaH6

The moisture percentage of the citrus exocarp and the yield of each of the extracts obtained from them were determined as follows:

$$\:Moisture\:\left(\%\right)\:=\frac{Dry\:exocarp\:\left(g\right)}{fresh\:exocarp\:\left(g\right)}*100$$

$$\:Yield\:\left(\%\right)\:=\frac{Final\:extract\:\left(g\right)}{Dry\:exocarp\:\left(g\right)}*100$$

### Colonization of *Ae. aegypti*

The colony was established from larvae collected through active searching in tanks, flowerpots, and tires in the municipality of Moniquirá-Colombia. The larvae were transported to the Medical and Forensic Entomology Laboratory at the Universidad Pedagógica y Tecnológica de Colombia, where the species was confirmed [37, 38]. The larvae were kept under insectary conditions (25 °C ± 3 °C, relative humidity 70% ± 15, photoperiod 12:12 h) and fed with dog food until they reached the adult stage, at which point they were provided with a 10% sucrose solution for sustenance. Additionally, the females were fed human blood twice a week. Eggs were collected on filter paper one day after blood ingestion [39].

### Adulticidal bioassays

From the concentrated extracts, solutions of 30, 60, 90, and 120 mg/mL were prepared using acetone. To evaluate adulticidal activity, the methodology of the World Health Organization was followed [40]. Briefly, 24 h before the bioassays, females from the colony were separated based on morphological characteristics [41]. During the assay, a 250 mL Schott bottle was impregnated with 1 mL of the extract solution. For each concentration, 25 adult females were introduced into the bottles. The number of dead mosquitoes was recorded for 72 h post-exposure (p.e), evaluating every hour during the first 12 h p.e, and then every 12 h thereafter. A bottle impregnated with acetone-water solutions, where water replaced the extract, was used as a negative control. A positive control used 1 mL of Baygon^®^ liquid insecticide. In all cases, the acetone was allowed to evaporate at room temperature before introducing the females. Each bioassay was performed in triplicate.

### Determination of chemical compounds present in the extracts

The identification of chemical compounds in the extracts was conducted using a GC VARIAN 3800 coupled to a VARIAN Saturn 2000 mass spectrometer, employing a Rtx-5 capillary column (30 m length x 0.25 mm internal diameter and 0.10 μm film thickness) with Helium as the carrier gas at a pressure of 15 Psi. The injector temperature was maintained at 250 °C, while the detector temperature was set at 230 °C. The column temperature program began at 60 °C for 1 min, then ramped up to 240 °C at a rate of 4 °C/min for 1 min, followed by a further increase to 280 °C at a rate of 10 °C/min for 1 min. Injection volume was 1.0 µL with a split ratio of 1:100. Analysis was performed in scan mode across the m/z 40–400 range, with a total run time of 40 min. Identification of components in the sample was achieved by comparing their mass fragmentation patterns with those available in the National Institute of Standards and Technology (NIST) library, version 17.

### Statistical analysis

The data obtained were analyzed using an Analysis of Variance (ANOVA) with a significance level of 0.05, followed by multiple comparison tests, including Tukey’s HSD and Bonferroni, to identify significant differences between groups. Additionally, the Lethal Concentrations 50 and 90 (LC_50_ and LC_90_) values were determined through PROBIT analysis. All statistical analyses were conducted using IBM SPSS Statistics software, version 27.

## Results

### Yields of the extracts obtained

The yield percentages of the extracts from the exocarp of *C. aurantium* and *C. latifolia* are presented in Table 2. As shown, the citrus species, the type of solvent, and the extraction time all influenced the yield percentage. Extracts from *C. aurantium* tend to have a higher yield compared to *C. latifolia* under most extraction conditions, this difference may be attributed to *C. aurantium* lower moisture content (22.1%) compared to *C. latifolia* (24.1%), implying a higher number of extractable compounds [42, 43]. Ethanol was the most effective solvent in terms of yield for both species and extraction times, while chloroform had an intermediate yield, and hexane was the least effective. This difference in yield may be attributed to the varying solvent polarity, as ethanol, with higher polarity, allowed the extraction of carbohydrates such as pectin, amylopectin, and cellulose present in the citrus exocarp [44], in addition to biologically active compounds. In contrast, chloroform, with intermediate polarity, extracted a smaller fraction of these compounds, and *n*-hexane, having the lowest polarity of the three solvents [45], extracted a minimal portion. An extraction time of 4 h generally results in higher yields compared to 6 h, possibly due to (i) the saturation of compounds, where extraction efficiency plateaus after a certain period, leading to a decrease in yield, and (ii) interactions between the compounds and the solvent, which may alter compound solubility and consequently impact extraction efficiency.

**Table 2:** Extract yield percentage

Code	% Yield
AuCl4	17.3
AuE4	24.1
AuH4	13.2
AuCl6	13.7
AuE6	15.4
AuH6	7.1
LaCl4	7.5
LaE4	14.4
LaH4	7.9
LaCl6	7.26
LaE6	10.2
LaH6	1.5

Au = *aurantium*, La = *latifolia*, Cl = Chloroform, E = Ethanol, H = *n*-hexane, 4 = 4 h of extraction and 6 = 6 h of extraction

### Adulticidal bioassays

**Fig. 1:**
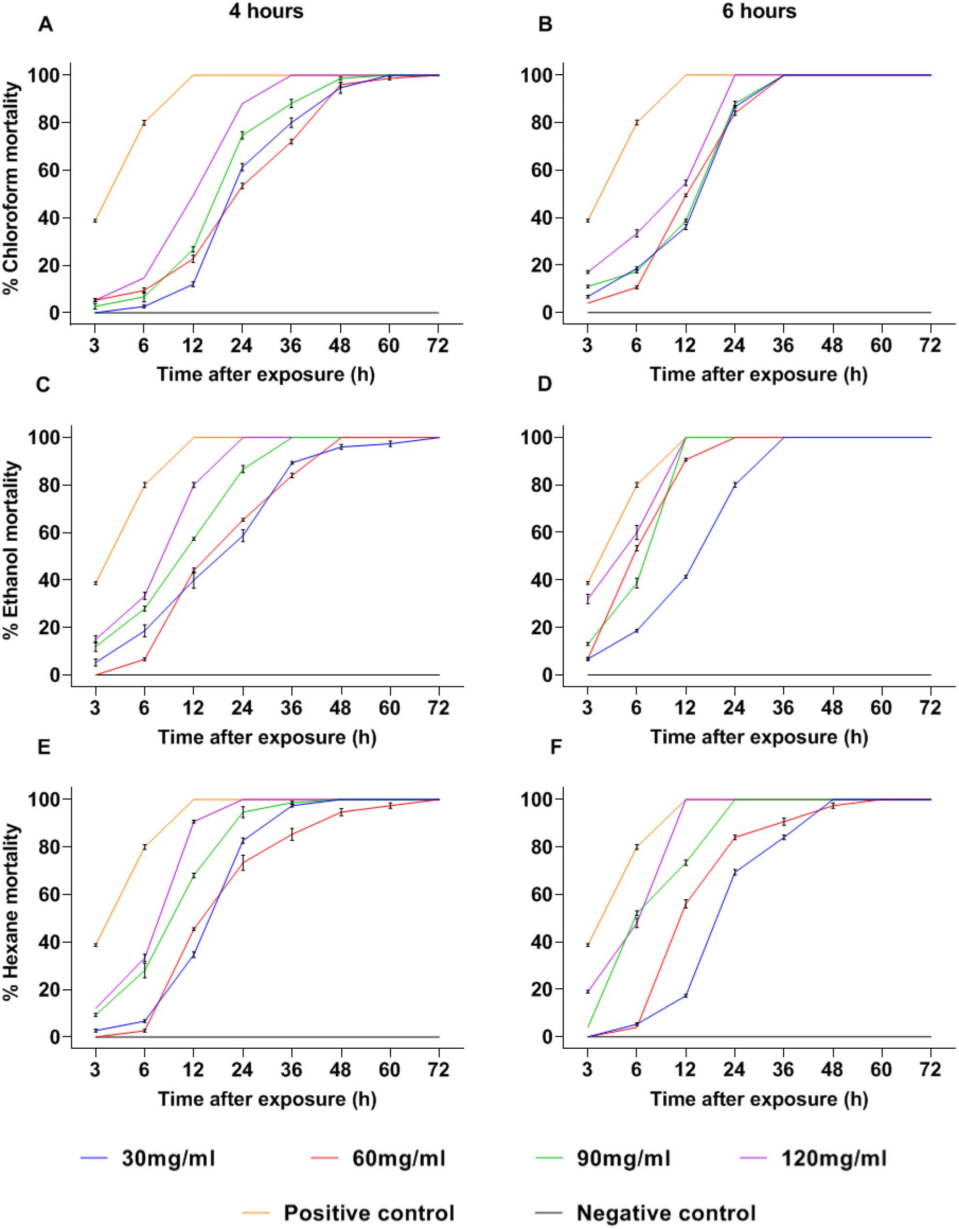
Percentage of Mortality with the *Citrus aurantium* extracts. (30, 60, 90 and 120 mg/mL correspond to the extract concentrations). (**A**), (**C**) and (**E**) = 4 h of extraction. (**B**), (**D**) and (**F**) = 6 h of extraction

When mosquitoes were exposed to extracts obtained from *C. aurantium*, the mortality percentages were low during the initial hours of exposure. However, the AuCl4 extract achieved a 100% mortality rate at 36 h p.e. with a concentration of 120 mg/mL (Fig. 1A). With the AuCl6 extract, using the same concentration, the time to reach total mortality decreased to 24 h p.e (Fig. 1B). Additionally, with the AuE4 and AuH4 extracts, 100% mortality was observed at 24 h p.e for the concentration of 120 mg/mL (Fig. 1C and E). For the AuE6 extract, 100% mortality was achieved at 12 h p.e with concentrations of 90 and 120 mg/mL (Fig. 1D). Meanwhile, with the AuH6 extract, it was observed that only the 120 mg/mL concentration achieved 100% mortality at the same time p.e (Fig. 1F). The analysis of variance revealed that the concentration, type of solvent and extraction time had a highly significant effect on the mortality of *Ae. aegypti* (*p* < 0.001). When post-hoc tests were carried out, the concentrations of 30 and 60 mg/mL did not show significant differences in mortality (*p* > 0.05), possibly because the lowest concentration of biologically active compounds was present under these conditions. which leads to its low activity against *Ae. aegypti*. Additionally, no significant differences in mortality (*p* > 0.05) were observed between extracts obtained with ethanol and hexane; however, during the bioassays, the ethanolic extracts demonstrated greater insecticidal activity.

**Fig. 2:**
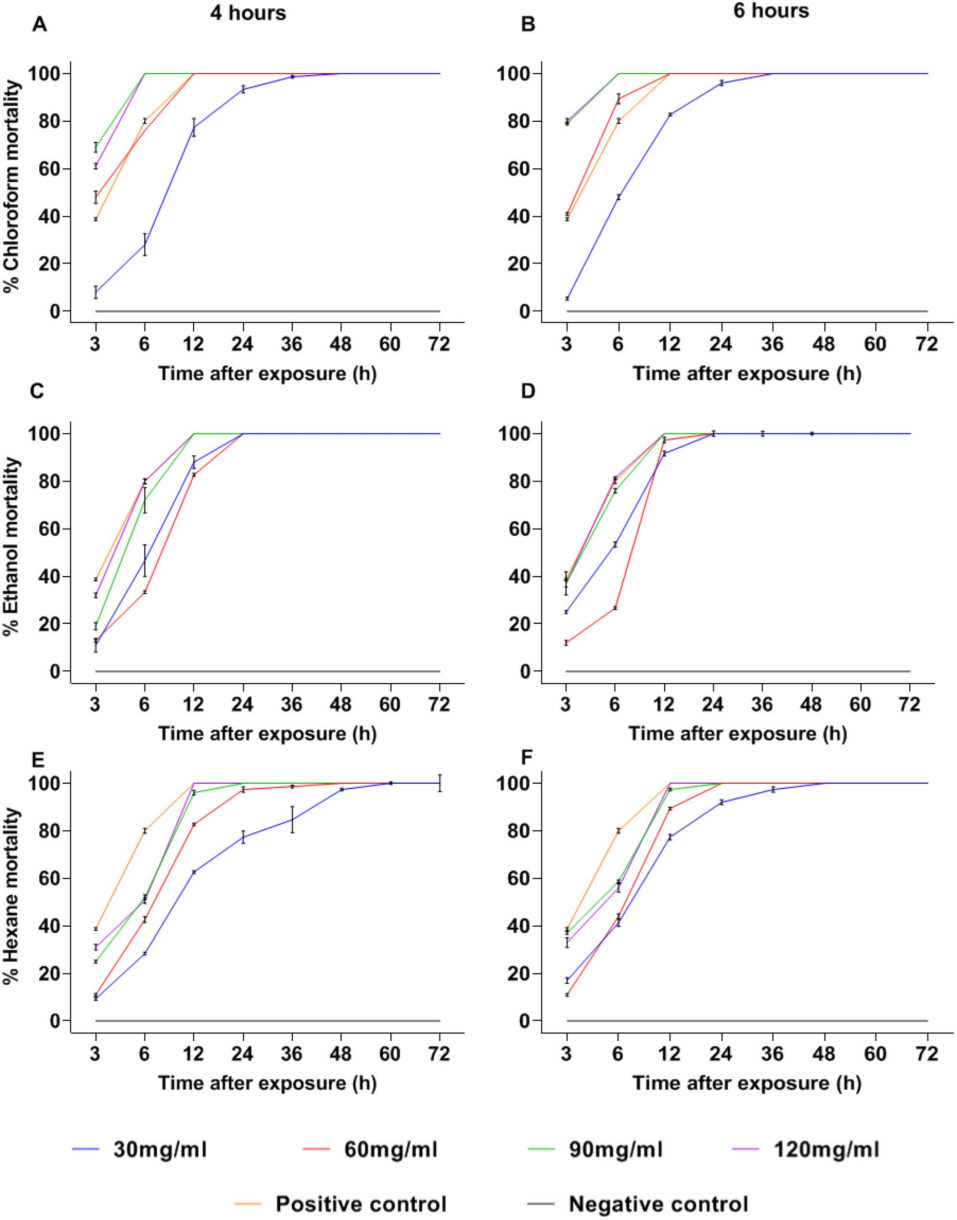
Percentage of Mortality with the *Citrus latifolia* extracts. (30, 60, 90 and 120 mg/mL correspond to the extract concentrations). (**A**), (**C**) and (**E**) = 4 h of extraction. (**B**), (**D**) and (**F**) = 6 h of extraction

In contrast to the results obtained with *C. aurantium*, extracts from *C. latifolia* showed high mortality within the first 3 h p.e when using the LaCl6 extract, reaching around 80% for both the 90 and 120 mg/mL concentrations and achieving 100% at 6 h p.e (Fig. 2A and B). Additionally, the LaE4 and LaE6 extracts showed 100% mortality at 12 h p.e with the 90 and 120 mg/mL concentrations (Fig. 2C and D). Meanwhile, with the 90 mg/mL concentration of the LaH4 and LaH6 extracts, 96% and 98% mortality were reached respectively at 12 h p.e. However, total mosquito mortality was achieved with the 120 mg/mL concentration at 12 h p.e (Fig. 2E and F).

According to the result of the analysis of variance, it was established that both the concentration and type of solvent had a highly significant effect on the mortality of *Ae. aegypti* (*p* < 0.001), while extraction time had less influence on the response variable (*p* = 0.018). Post-hoc tests revealed no significant differences in mortality between the 90 and 120 mg/mL concentrations across all extracts (*p* > 0.05). Moreover, mortality was significantly influenced by the type of solvent used for extraction (*p* < 0.05).

In general, the results of this study reveal that the extracts obtained from the exocarp of *C. aurantium* and *C. latifolia* exhibit high adulticidal activity against *Ae. aegypti*. Notably, *C. latifolia* extracts demonstrated faster mortality induction, achieving 100% mortality within 6 to 12 h p.e. depending on the solvent and concentration used. In contrast, *C. aurantium* extracts also reached total mortality, albeit over a longer time of 12 to 36 h p.e. Among the *C. aurantium* extracts, those obtained with ethanol and hexane were the most effective, achieving 100% mortality at 24 h p.e. at a concentration of 120 mg/mL. Meanwhile, *C. latifolia* extracts showed superior performance, achieving complete mortality within 6 to 12 h p.e., particularly when chloroform was used as solvent. These findings connect directly with the aim of this research emphasizing the fast and effective action of these extracts and highlighting their potential as an environmentally sustainable alternative for vector control.

Biologically it was established that chloroform extracts, mainly the one obtained at 6 h, exhibited greater activity against the vector from the first hours of evaluation.

### Determination of LC_50_ and LC_90_

When the lethal concentrations of the *Citrus aurantium* extracts were analyzed in relation to the polarity of the solvents and the extraction time, the best results were obtained with AuE6, showing an LC_50_ of 32.2 ± 0.31 mg/mL at 12 h p.e. These results demonstrated moderate effectiveness compared to *C. latifolia* extracts, where the most effective outcome of the study was achieved with LaCl6 exhibiting an LC_50_ of 9 ± 0.25 mg/mL at 12 h p.e (Table 3).

**Table 3:** LC_50_ and LC_90_ (mg/mL) values of citrus extracts against *Ae. aegypti* mosquitoes 6 and 12 h post-exposure

Code	Post-exposition time (6 h)		Post-exposition time (12 h)	
	CL_50_	CL_90_	CL_50_	CL_90_
AuCl4	1047.7 ± 0.23	12508.4 ± 0.17	156.6 ± 0.36	880.9 ± 0.35
AuCl6	2102 ± 0.25	342695.1 ± 0.45	111.8 ± 0.35	16266.5 ± 0.36
AuE4	551.5 ± 0.45	14270.3 ± 0.42	53 ± 0.25	331 ± 0.25
AuE6	88.7 ± 0.59	571 ± 0.40	32.2 ± 0.31	61.6 ± 0.31
AuH4	238.2 ± 0.23	1072.2 ± 0.40	61.1 ± 0.26	186.9 ± 0.25
AuH6	124.2 ± 0.40	322.5 ± 0.23	51.8 ± 0.29	94.8 ± 0.17
LaCl4	38.9 ± 0.25	68.8 ± 0.31	12.8 ± 0.35	38.4 ± 0.46
LaCl6	30.1 ± 0.23	60.1 ± 0.45	9 ± 0.25	33.5 ± 0.23
LaE4	48.3 ± 0.30	289.9 ± 0.35	19.7 ± 0.21	73.8 ± 0.23
LaE6	63.1 ± 0.21	168.5 ± 0.50	27.8 ± 0.12	49.5 ± 0.30
LaH4	67.7 ± 0.31	139.9 ± 0.35	51 ± 0.25	78.5 ± 0.35
LaH6	66.5 ± 0.42	3351.7 ± 0.17	17.9 ± 0.21	56.3 ± 0.30

Au = Aurantium, La = Latifolia, Cl = Chloroform, E = Ethanol, H = Hexane, 4 = 4 h of extraction y 6 = 6 h of extraction

### Identification of compounds by GC-MS

**Fig. 3:**
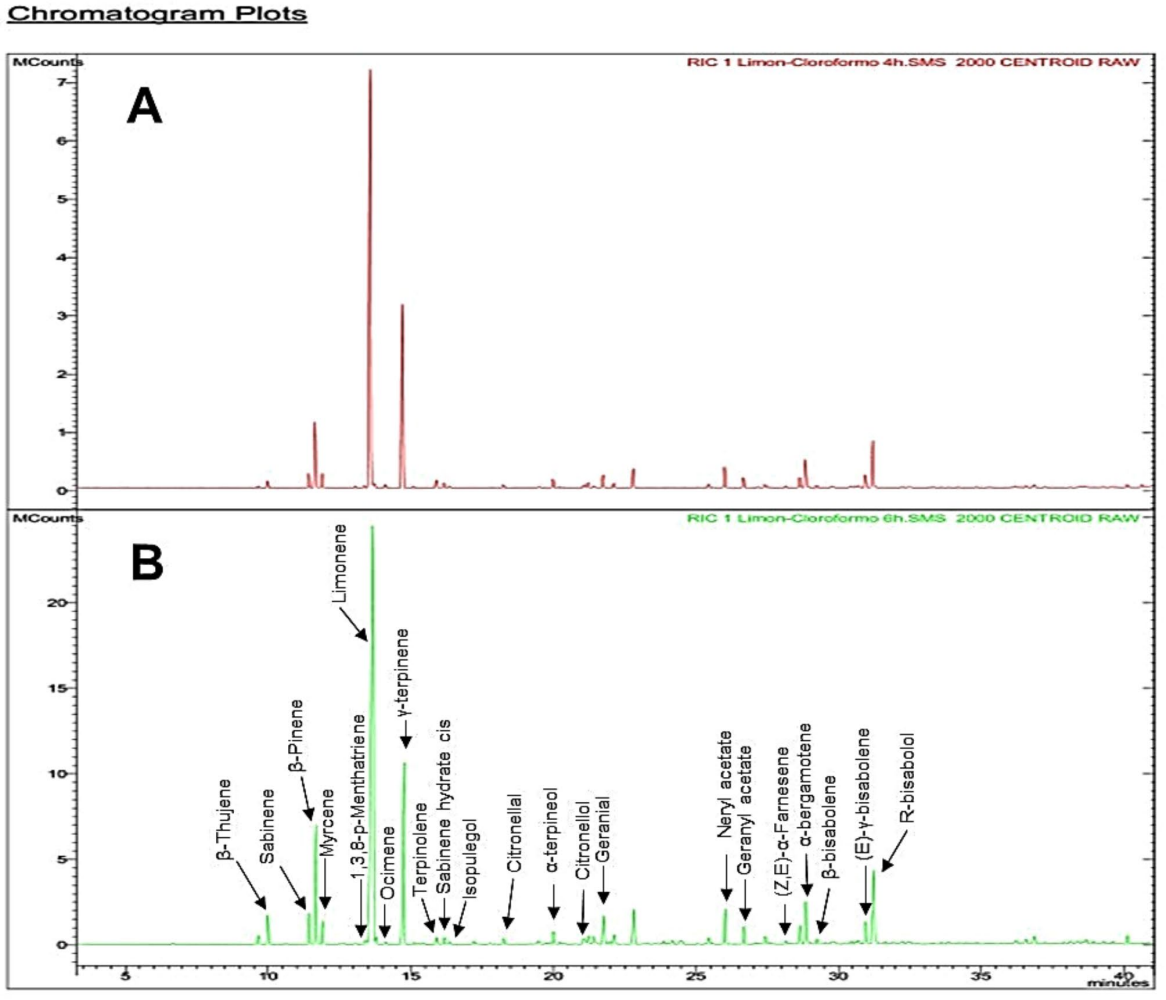
Main compounds identified in the chloroform extracts of the exocarp of *C. latifolia* by GC-MS. (**A**) 4 h of extraction, (**B**) 6 h of extraction

Due to the lower lethal concentrations required to achieved 100% mortality of *Ae. aegypti* with the LaCl4 and LaCl6 extracts, these were selected for compound identification. Figure 3 shows the chromatograms, and Table 4 lists the identified components for each extract. The major component was R-limonene, and the concentration of most identified components increased when the extraction time was 6 h.

**Table 4:** Chemical composition in the LaCl4 and LaCl6 extracts identified by GC-MS

TR (min)	Chemical compound	% LaCl4	% LaCl6
10	β-Thujene	0.72	1.75
11.4	Sabinene	1.4	2.04
11.7	β-Pinene	6.49	8.25
11.9	Myrcene	1.43	1.32
13.4	1.3.8-p-Menthatriene	0.21	0.3
13.6	R-limonene	46.74	48.7
14.1	Ocimene	0.36	0.17
14.7	γ-terpinene	19.91	13.82
15.9	Terpinolene	0.81	0.45
16.2	Sabinene hydrate cis	0.54	0.42
16.4	Isopulegol	0.23	0.17
18.3	Citronellal	0.35	0.35
20	α-terpineol	0.97	0.75
21.1	Citronellol	0.34	0.47
21.2	ni	0.54	0.46
21.7	Geranial	1.41	1.86
22.1	ni	0.54	0.57
22.8	ni	2.06	2.41
26	Neryl acetate	2.34	2.27
26.7	Geranyl acetate	1.12	1.14
28.2	(Z.E)-α-Farnesene	0.21	0.18
28.6	ni	1.26	1.33
28.8	α-bergamotene	2.9	3.13
29.2	β-bisabolene	0.27	0.34
30.9	(E)-γ-bisabolene	1.52	1.65
31.2	R-bisabolol	5.33	5.69

TR: retention time; ni = not identified

## Discussion

The effectiveness of ethanol as a solvent can be attributed to its higher polarity, which facilitated the extraction of carbohydrates such as pectin, amylopectin, and cellulose, from the citrus exocarp, as well as biologically active compounds [44]. Chloroform, with intermediate polarity, extracted a smaller fraction of these compounds, while hexane, the least polar of the three solvents [45], extracted only a minimal portion. Regarding extraction time, it was observed that a time of 4 h generally results in higher yields compared to 6 h. This could be explained by two factors: (a) compound saturation, where after a certain period, the rate of compound extraction stabilizes, leading to a decrease in yield, and (b) interaction between compounds present in the sample and the solvent, which can influence compound solubility and, consequently, extraction efficiency.

In general, it was observed that the results of the bioassays conducted with the exocarp extracts of *C. aurantium* and *C. latifolia* exhibited dose- and time-dependent mortality percentages, with the highest mortality observed at the highest concentrations (90 and 120 mg/mL). This suggests a relationship between the biological activity of the compounds in the extracts and their effect on the viability of *Ae. aegypti*. In our study, the high adult mortality values and LC_50_ obtained with *C. aurantium* (AuE6) of 32.2 mg/mL at 12 h p.e and with *C. latifolia* (LaCl6) with an LC_50_ of 9 mg/mL at 12 h p.e, differ from those previously reported by Leyva et al., [46], where the adulticidal effect of essential oil from *C. aurantium* extracted from the fruit epicarp, was evaluated using impregnated bottle assays. However, no adulticidal activity was observed at a concentration of 60 mg/mL. Similar results were reported by Sarma [47], who evaluated the adulticidal activity of essential oil from the exocarp of *C. aurantifolia*, finding low mortality rates, thus failing to establish LC_50_ values at concentrations of 0.1 and 1 mg/mL. Conversely, this oil showed ovicidal activity with an LC_50_ of 0.017 mg/mL and larvicidal activity with an LC_50_ of 0.128 mg/mL at 24 h, demonstrating toxic action on the immature stages of *Ae. aegypti*. Additionally, methanolic extracts of *C. aurantifolia* leaves were evaluated on mosquito larvae, but not on adults, showing toxicity with an LC_50_ at 24 h of 2.2 mg/mL and an LC_95_ of 3.66 mg/ [48]. These findings highlight the significant influence of extraction method and duration on mortality values in *Ae. aegypti*. Furthermore, the species of citrus used can greatly contribute to the variation in LC_50_ values and, therefore, in mosquito mortality results.

On the other hand, when comparing the results of the present study with other natural extracts of non-citrus origin, similar LC50 values with adulticidal activity against *Ae. aegypti* have been reported. For instance, Ninditya et al. (2020) obtained LC50 values of 11.35 mg/mL using ethanol-extracted Artemisia vulgaris leaves [49]. Similarly, Pratheeba et al. (2019) reported an LC50 of 11.810 mg/ mL using hexane-extracted *Pavetta tomentosa* leaves [50]. In contrast, Fernandes et al. (2021) obtained extracts from the leaves and stems of *Helicteres velutina*, which showed LC50 values of 0.74 mg/mL and 8.01 mg/mL when dichloromethane and hexane were used as solvents, respectively. In line with these findings, the LC50 values obtained with *C. latifolia* extracts are promising for the development of a citrus-based bioinsecticide [51].

It is worth noting that the results in this study were obtained under controlled conditions, where variables such as temperature, humidity, and photoperiod are tightly regulated in the laboratory. This control limits the representation of the real environmental fluctuations observed in field conditions. Additionally, *Ae. aegypti* populations in the field may exhibit significant genetic variability, which could influence their susceptibility to the citrus extracts tested under our controlled conditions. Moreover, factors such as extract concentration and exposure time required to achieve high adulticidal efficacy may differ in the field due to environmental conditions and local ecological dynamics. Therefore, it is necessary to complement laboratory assays with evaluations in natural settings.

The major component, R-limonene, is a natural compound present in citrus fruits, with known insecticidal activity against *Ae. aegypti* [52]. The action of this compound is attributed to the inhibition of acetylcholinesterase, a crucial enzyme in the nervous system of insects [53]. The slight increase in R-limonene concentration observed in the LaCl6 extract may have contributed to the improved insecticidal effect. Other identified compounds also increased their concentration when the extraction time was prolonged and may have also contributed to the increased mortality of *Ae. aegypti*. For instance, β-thujene showed the highest increase (2.4 times) when comparing the extraction time from 4 to 6 h (Table 4). This monoterpene has been recognized as an active insecticidal component against *Ae. aegypti* and, due to its hydrophobic nature allows it to penetrate the insect cuticle, increasing its toxicity compared to polar compounds [54]. β-thujene is present in essential oils from *Origanum vulgare* L. and *Thymus vulgaris* L., which have demonstrated significant adulticidal activity against mosquitoes, with LC_50_ values of 14.3 and 11.7 mg/mL, respectively [55]. It has also been found in essential oils of *Lavender angustifolia*, as well as in species of the Asteraceae and Myrtaceae families, which have shown larvicidal activity against *Ae. aegypti* [56]. The increase in the concentration of compounds such as R-limonene and β-thujene with extended extraction time likely contributed to the enhanced insecticidal effect of the extracts. R-limonene, with its ability to inhibit acetylcholinesterase, and β-thujene, with its high hydrophobicity and ability to penetrate the insect cuticle, are key contributors to the observed increase in toxicity [52, 54].

Sabinene was the second compound to show the most significant increase (1.5 fold) when comparing extraction times. This compound has been identified by various authors in the essential essential oils of *Myristica fragrans*,* Zanthoxylum schinifolium*, and *Launaea taraxacifolia*, all of which have demonstrated high toxicity against *Ae. aegypti* in both larval and adult stages [54, 57]. Although few studies specifically attribute the action of sabinene against *Ae. aegypti*, its toxicity has also been investigated in other pest species, such as the corn and rice weevils (*Sitophilus zeamais* and *Sitophilus oryzae*), with positive results [57, 58].

Other compounds reported to have insecticidal activity against *Ae. aegypti*, as well as other medically and agriculturally significant species, and which also increased in concentration with extended extraction time to 6 h in this study, include 1,3,8-p-Menthatriene [59, 60], Citronellol [61, 62], β-Pinene [63, 64], Geranial [65], and β-Bisabolene [66].

In line with the aforementioned findings, the high mortality of *Ae. aegypti* observed with the LaCl6 extract is likely due to the presence of components extracted via reflux using chloroform as the solvent. Additionally, a synergistic interaction among these components may enhance their insecticidal efficacy. Similar results have been reported by Owolabi [58] when evaluating the insecticidal activity of the essential oil of *Launaea taraxacifolia*, whose main components were limonene and sabinene.

## Conclusions

The yield of the extracts depends on the type of solvent, extraction time, and the moisture content of the citrus exocarp. A lower yield was obtained as the solvent’s polarity decreased. Prolonged extraction time can decrease yield due to saturation and interactions between compounds. Additionally, lower moisture content in the plant material facilitates greater compound extraction. Adulticidal bioassays using extracts from the exocarp of both *C. aurantium* and *C. latifolia* revealed a dose- and time-dependent response on *Ae. aegypti* mortality, indicating a direct relationship between the biological activity of the compounds in the extracts and their lethality as exposure time increased. Furthermore, an important relationship was established between citrus species, solvent polarity, and extraction time with lethal concentrations, showing that vector susceptibility was highest when using the exocarp extract of *C. latifolia* obtained with chloroform and a 6 h extraction time (LaCl6). The main component in this extract was R-limonene, and the high mortality of *Ae. aegypti* achieved was likely due to the combined action of this compound with other components, primarily β-thujene, sabinene, and pinene. The results of this study highlight the effectiveness of using citrus extracts for controlling *Ae. aegypti*. Consequently, a second phase of the project will be conducted to determine the susceptibility of the mosquito in field using the chloroform extract of *C. latifolia*, with the aim of developing a commercial bioinsecticide.

## Data Availability

The datasets generated during and/or analyzed during the current study are available from the corresponding author upon reasonable request.
