# Correction: *Citrus aurantium* L. and *Citrus latifolia* extracts as alternative control agents for *Aedes aegypti* (Diptera: Culicidae)

**DOI:** 10.1186/s40659-025-00631-4

**Published:** 2025-07-18

**Authors:** Andrea Martínez Gordon, Alejandro Figueredo López, Ingrid Dayana Jiménez, Laura Barrera Martínez, Oscar H. Pardo Cuervo, Nidya Alexandra Segura Guerrero

**Affiliations:** 1 Grupo de Investigación en Ciencias Biomédicas UPTC/GICBUPTC, Universidad Pedagógica y Tecnológica de Colombia - UPTC, Tunja, Boyacá Colombia; 2 Grupo de Investigación Catálisis, Universidad Pedagógica y Tecnológica de Colombia - UPTC, Tunja, Boyacá Colombia

**Correction: Biological Research (2025) 58:41**

10.1186/s40659-025-00600-x

In this article the author’s name Ingrid Dayana Jiménez was incorrectly written as Ingrid Dayana Jiménez López.

The original article has been corrected.

